# Hypoxia in grape berries: the role of seed respiration and lenticels on the berry pedicel and the possible link to cell death

**DOI:** 10.1093/jxb/ery039

**Published:** 2018-03-06

**Authors:** Zeyu Xiao, Suzy Y Rogiers, Victor O Sadras, Stephen D Tyerman

**Affiliations:** 1 The Australian Research Council Training Centre for Innovative Wine Production, The University of Adelaide, Glen Osmond, SA, Australia; 2 School of Agriculture, Food and Wine, Waite Research Institute, The University of Adelaide, PMB1, Glen Osmond, SA, Australia; 3 NSW Department of Primary Industries, Wagga Wagga, NSW, Australia; 4 National Wine and Grape Industry Centre, Charles Sturt University, Wagga Wagga, NSW, Australia; 5 South Australian Research & Development Institute, Waite Research Precinct, Urrbrae, SA, Australia

**Keywords:** Grape berry, lenticels, micro-CT, oxygen sensor, pedicel, programmed cell death, respiration, seed respiration, temperature, *Vitis vinifera*

## Abstract

Mesocarp cell death (CD) during ripening is common in berries of seeded *Vitis vinifera* L. wine cultivars. We examined if hypoxia within berries is linked to CD. The internal oxygen concentration ([O_2_]) across the mesocarp was measured in berries from Chardonnay and Shiraz, both seeded, and Ruby Seedless, using an oxygen micro-sensor. Steep [O_2_] gradients were observed across the skin and [O_2_] decreased toward the middle of the mesocarp. As ripening progressed, the minimum [O_2_] approached zero in the seeded cultivars and correlated to the profile of CD across the mesocarp. Seed respiration declined during ripening, from a large proportion of total berry respiration early to negligible at later stages. [O_2_] increased towards the central axis corresponding to the presence of air spaces visualized using X-ray micro-computed tomography (CT). These air spaces connect to the pedicel where lenticels are located that are critical for berry O_2_ uptake as a function of temperature, and when blocked caused hypoxia in Chardonnay berries, ethanol accumulation, and CD. The implications of hypoxia in grape berries are discussed in terms of its role in CD, ripening, and berry water relations.

## Teaser

Grape berry internal oxygen concentration is dependent upon lenticels on the pedicel, and cell death in the mesocarp is correlated to hypoxia during ripening.

## Introduction

Onset and rate of cell death (CD) in berry mesocarp of *Vitis vinifera* L. are genotype dependent, and modulated by temperature and drought (Krasnow *et al.*, 2008; Tilbrook and Tyerman, 2008; Fuentes *et al.*, 2010; Bonada *et al.*, 2013a, b). Evolutionarily, CD may have been selected as a trait favouring seed dispersal (Hardie *et al.*, 1996). It correlates with berry dehydration (Fuentes *et al.*, 2010; Bonada *et al.*, 2013*a*), a common phenomenon in warm wine-growing regions, and is partially distinct from other forms of ‘berry shrivel’ (Bondada and Keller, 2012; Keller *et al.*, 2016). Berry dehydration associated with CD is common in Shiraz (Syrah), resulting in increased sugar concentration (Rogiers *et al.*, 2004; Sadras and McCarthy, 2007; Caravia *et al.*, 2016). It is also associated with altered chemical composition (Šuklje *et al.*, 2016) and sensory characteristics (Bonada *et al.*, 2013*b*).

Grape berries are non-climacteric, though ethylene may still play a role (Bottcher *et al.*, 2013). However, the onset of ripening is associated with the accumulation of hydrogen peroxide (H_2_O_2_) in skin of Pinot Noir berries (Pilati *et al.*, 2014). Although H_2_O_2_ was considered a harmless signal, Pinot Noir berries also show up to 50% CD (Fuentes *et al.*, 2010). Accumulation of H_2_O_2_ is also characteristic of plant tissues exposed to hypoxia or anoxia (Blokhina *et al.*, 2001; Fukao and Bailey-Serres, 2004). The grape berry respiratory quotient increased during ripening (Harris *et al.*, 1971) associated with increased ethanolic fermentation (Terrier and Romieu, 2001; Famiani *et al.*, 2014). Other fruit also show restricted aerobic respiration and fermentation (Hertog *et al.*, 1998). Ethanolic fermentation contributes to maintain cell function under O_2_-limiting conditions provided sugars are available. Interestingly, both H_2_O_2_ and ethylene have been implicated in its regulation (Fukao and Bailey-Serres, 2004).

Hypoxia-induced oxidative stress decreases lipid and membrane integrity (Blokhina *et al.*, 2001), the latter being clearly evident in most wine grape berries by vitality stains (Tilbrook and Tyerman, 2008). Increased CD in Shiraz grapes is reflected by decreased extracellular electrical resistance indicating electrolyte leakage (Caravia *et al.*, 2015). This leakage corresponds to the accumulation of potassium in the extracellular space of Merlot berries (Keller and Shrestha, 2014), a cultivar that also displayed CD (Fuentes *et al.*, 2010). O_2_ deprivation diminishes intracellular energy status that reduces cell vitality in non-photosynthetic organs, as exemplified by roots under flooding or waterlogging (Voesenek *et al.*, 2006). Although grape berries show some photosynthesis in early stages of development (Ollat and Gaudillère, 1997), during ripening photosynthetic pigments and nitrogen content are reduced and atmospheric CO_2_ is not fixed, while re-fixation of respiratory CO_2_ declines (Palliotti and Cartechini, 2001).

Shiraz berry CD can be accelerated by water stress and elevated temperature (Bonada *et al.*, 2013*b*). There are increasing frequencies and intensities of heat waves and drought events globally including Australia (Alexander and Arblaster, 2009; Perkins *et al.*, 2012), and the warming trend is predicted to have adverse effects on grapevines (Webb *et al.*, 2007) and berry quality (Fuentes *et al.*, 2010; Bonada and Sadras, 2015; Caravia *et al.*, 2016). Higher temperature increases demand for O_2_ to support increased oxidative respiration (Kriedemann, 1968). Meanwhile, O_2_ diffusion into the berry may be limited by decreased gas exchange across the berry skin during ripening, as judged by declining transpiration (Rogiers *et al.*, 2004; Scharwies and Tyerman, 2017) and/or changes in berry internal porosity during ripening. Lenticels on the skin of potato tubers are the main channel for O_2_ uptake for respiration (Wigginton, 1973), and the phellem–lenticel complex of woody roots and trunks regulates O_2_ exchange (Lendzian, 2006). In the grape berry, the small number of stomata on skin develop into non-functional lenticels occluded with wax (Rogiers *et al.*, 2004), but lenticels are very prominent on the pedicel (Becker *et al.*, 2012).

Wine grape cultivars are seeded, and have been selected for wine-related attributes, whereas table grape cultivars have been selected for turgor maintenance, and markets increasingly demand seedless fruit; these differences in selective pressures between wine and table grape cultivars have led to differences in the dynamics of water during berry ripening (Sadras *et al.*, 2008). Table grape seedless cultivars show little or no CD well into ripening (Tilbrook and Tyerman, 2008; Fuentes *et al.*, 2010). Although lignification of seeds is complete before berries begin to ripen (Cadot *et al.*, 2006), oxidation of seed tannins is sustained (Ristic and Iland, 2005) and is concurrent with oxidation of phenolic compounds such as flavan-3-ol monomers and procyanidins (Cadot *et al.*, 2006). Lignin polymerization requires consumption of O_2_ and generation of H_2_O_2_ for the final peroxidase reaction (Lee *et al.*, 2013), and this, with oxidation of tannins, could put additional stress on the mesocarp in seeded cultivars. Phenolic compounds can also act as reactive oxygen species (ROS) scavengers (Blokhina *et al.*, 2003). In grape, biosynthesis of procyanidins coincides with the initial rapid period of growth (Coombe, 1973), and flavan-3-ol accumulated during early ripening (Cadot *et al.*, 2006). Taken together, seed respiration and maturation deserve consideration in understanding mesocarp CD.

In this study, we test the hypothesis that hypoxia {i.e. below normoxia as 20.95% air O_2_ concentration [O_2_] (Sasidharan *et al.*, 2017)} occurs within the grape berry during ripening and that this may be correlated with CD in the pericarp. We compared the patterns of CD and [O_2_] profiles across the berry flesh of two wine, seeded cultivars, Chardonnay and Shiraz, and a seedless table grape cultivar, Ruby Seedless. Respiratory demand of seeds and berries were measured for different ripening stages and different temperatures. The diffusion pathway of O_2_ supply was assessed through examination of the role of lenticels in the berry pedicel and air space estimates using X-ray micro-computed tomography (micro-CT) of single berries.

## Materials and methods

### Berries from vineyards

Details of sources of berries, vine age, sampling times, and corresponding measurements are listed in Table 1. Berries from the Waite Campus (34°58'04.8''S, 138°38'07.9''E) vineyards were sampled over the 2014–2015, 2015–2016, and 2016–2017 seasons. Mature Shiraz, Chardonnay, and Ruby Seedless vines on their own roots were grown under standard vineyard management with vertical shoot positioning, spur pruning (two buds), and drip irrigation on dark brown clay soils with shale fragments, grading into red-brown mottled clay; overlying olive-brown mottled cracking clay (Du Toit, 2005). Rows (3 m spacing) were north–south oriented. Three replicates each consisted of two vines per replicate for Shiraz and three vines per replicate for Chardonnay. Ten random clusters (combination of proximal and distal) were labelled within each replicate, and 20 berries (two from each cluster, randomly located within the cluster) per replicate were excised at the pedicel–rachis junction with sharp scissors at each sampling date between 09.00 h and 11.00 h. Ruby Seedless grapes were sampled from three vines with five clusters labelled for sampling on each vine, and 20 berries were sampled from each vine. Timing of sampling during berry development was measured as days after anthesis (DAA, 50% of caps fallen from flowers). Berries were placed in sealed plastic bags into a cooled container, and taken to the laboratory, stored at 4 °C in the dark, and tested within 48 h of sampling.

**Table 1.** Summary of berry source and traits measured

Source of berries	**Cultivar**	**Plant date**	**Season**	**Traits**	**Sampling time**	**Replication**
**Waite vineyards**	Chardonnay	1995	2015–2016; 2016–2017	O_2_ profile Berry and seed respiration Micro-CT O_2_ profile when N_2_ applied Respiration (lenticel blockage) Respiration (temperature change)	87, 104, 136 DAA 63, 122 DAA 98, 154 DAA 90 DAA 86 DAA 76, 120 DAA	3 reps, 3 berries per rep 3 reps, 1 berry per rep
	Shiraz	1993	2014–2015; 2016–2017	O_2_ profile Respiration (lenticel blocked) Respiration (temperature change)	85, 114 DAA 77 DAA 71, 113 DAA	3 reps, 3 berries per rep
	Ruby seedless	1992	2016–2017	O_2_ profile O_2_ logging	91, 132 DAA 132 DAA	3 reps, 3 berries per rep
**Growth chamber, cuttings from Waite vineyard**	Chardonnay	2015	2015	Lenticel	At veraison	5 berries
	Shiraz	2015	2015	Lenticel	At veraison	5 berries
**Growth chamber, rootlings from Yalumba**	Chardonnay	2017	2017	Lenticel blockage on vines (O_2_, cell vitality, ethanol)	3, 5, 7, 10, 12, 14, 18, and 20 d after blockage	2 or 3 berries

### Berries from pot-grown vines

Shiraz and Chardonnay cuttings were taken from the Waite vineyards in April 2015 and propagated after storage at 4 °C in the dark for ~2 weeks. The propagation method and vine nutrition management were based on Baby *et al.* (2014). Briefly, after roots were initiated in a heated sand bed in a 4 °C cold room for 8 weeks, and after the root length reached ~6 cm, cuttings were transferred into a vermiculite:perlite (1:1) mixture in 12 cm pots. Pots were placed in a growth chamber with a 16 h photoperiod, 400 μmol photons m^–2^ s^–1^) at the plant level, 27 °C day/22 °C night, and 50% humidity. Pots were irrigated with half-strength Hoagland solution (Baby *et al.*, 2014). Fruitful vines at stage EL-12 (Coombe, 1995) were then transferred into a University of California (UC) soil mix: 61.5 litres of sand, 38.5 litres of peat moss, 50 g of calcium hydroxide, 90 g of calcium carbonate, and 100 g of Nitrophoska^®^ (12:5:1, N:P:K plus trace elements; Incitec Pivot Fertilisers, Southbank, Vic., Australia), per 100 litres at pH 6.8, in 20 cm diameter (4 litre) pots irrigated with water thereafter. Five berries (each from three different vines) of each cultivar were used for light stereomicroscopy.

Chardonnay rootlings were obtained from Yalumba Nursery in April 2017 and planted with UC mix soil and in the same growth chamber with the same growth conditions as above. Seven vines, each with one cluster, were used for O_2_ diffusion experiments.

### [O_2_] profiles in berries

**Fig. 1.**
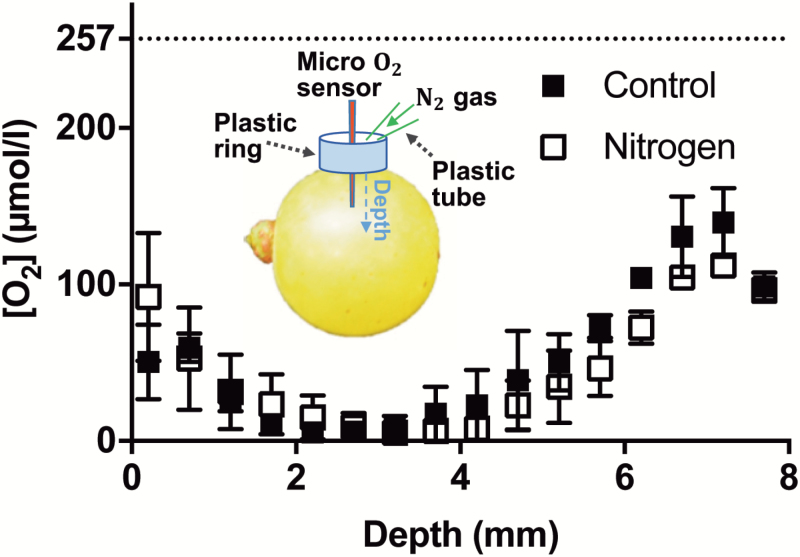
[O_2_] profiles of Chardonnay berries (90 DAA in the 2016–2017 season, Waite vineyards) measured with and without N_2_ gas applied at the entry point during measurement. Inset: experimental set-up for measuring berry [O_2_] profiles (not to scale). The O_2_ sensor (tip diameter 25 µm) was inserted at the equator of the berry and moved inwards to the centre approximately across the radius. Around the entry of the sensor, a plastic ring was sealed and glued to the berry, to contain nitrogen gas gently flowing on to the entry point of the sensor. Data are means ±SE, *n*=3. Two-way ANOVA (repeated measures) showed that depth accounted for 68.73% of total variation (*P*<0.0001), treatments accounted for 0.55% of total variation (*P*=0.26), and interaction accounted for 3.72% of total variation (*P*=0.87).

Berry [O_2_] was measured using a Clark-type O_2_ microelectrode with a tip diameter of 25 µm (OX-25; Unisense A/S, Aarhus, Denmark). The microelectrodes were calibrated in a zero O_2_ solution (0.1 M NaOH, 0.1 M C_6_H_7_NaO_6_) and an aerated Milli-Q water (272 µmol l^–1^ at 22 °C), as 100% O_2_ solution. Individual berries (equilibrated to room temperature) were secured on the motorized micromanipulator stage. To aid the penetration of the microelectrode into the berry skin, the skin was pierced gently with a stainless-steel syringe needle (19 G), to a depth of 0.2 mm, at the equator of the berry. The microsensor was positioned in the berry through this opening and [O_2_] profiles were taken with depth towards the centre of the berry. For Shiraz, measurements were taken from 0.2 mm to 1.5 mm under the skin at 0.1 mm increments. The electrode was not moved beyond this point to avoid damaging the tip against a seed. For Ruby Seedless, where seeds were not present, and Chardonnay grapes, where there were no seeds present or the position of the seeds could be determined through the semi-transparent skin, measurements were taken at 0.5 mm intervals from 0.2 mm under the skin to the berry centre. Each measurement was applied for a 10 s duration at each depth. Between each position, a 20 s waiting time was applied to ensure stable signals. To test whether puncturing of the skin by the needle and insertion of the microelectrode contaminated the berry internal O_2_ by the surrounding air, a plastic ring was placed around the insertion site and a gentle stream (250 ml min^–1^) of nitrogen gas was applied to the insertion point while obtaining the O_2_ readings (Fig. 1A).These readings were compared with those where no nitrogen gas was applied.

The O_2_ readings were recorded using the Unisense Suite software (Unisense A/S). Three berries were measured for each biological replicate. Means and SE of each step (*n*=3) were calculated and [O_2_] profiles were compiled using GraphPad Prism 7 (GraphPad Software Inc., La Jolla, CA, USA). Following the O_2_ measurements, berry temperature was recorded using an IR thermometer (Fluke 568, Fluke Australia Pty Ltd, NSW, Australia) with a type-K thermocouple bead probe (Fluke 80PK-1). Berry diameters at the equator were measured with a digital calliper. [O_2_] and respiration (see below) were measured under dim room lighting, <1 μmol photon m^–2^ s^–1^. Berry vitality was determined (see below) and total soluble solids (TSS) of the juice from individual berries were determined using a digital refractometer (Atago, Tokyo, Japan) as an indicator of berry maturity.

### Testing the role of pedicel lenticels

[O_2_] was measured as above but with the probe stationary at ~2 mm from the pedicel along the berry central axis. After a stable reading was obtained, N_2_ gas (250 ml min^–1^) was then applied over the pedicel in order to test the contribution of pedicel lenticels to O_2_ diffusion into the berry.

### Berry and seed respiratory O_2_ consumption

A Clark-type oxygen microsensor OX-MR and the MicroRespiration System (Unisense A/S) were used for berry and seed respiration measurements. A replicate consisted of nine berries. The measuring chamber was filled with aerated MilliQ water, constantly stirred, and was maintained at 25 °C in a water bath. After the measurement of whole berry respiration, seeds of the nine berries were extracted and the seed respiration rate measured using the same apparatus. Changes in the chamber’s water [O_2_] were monitored for at least 15 min, with readings taken every 5 s in order to determine a steady respiration rate from the slope of the decline in [O_2_].

Respiration was also measured for Shiraz and Chardonnay berries before and after the pedicels were covered with silicone grease (SGM494 silicone grease, ACC Silicones Limited, Bridgewater, UK), which was known to restrict berry pedicel water uptake (Becker *et al.*, 2012), at 20 °C and 40 °C. Another batch of nine Chardonnay berries was used to determine the respiratory contribution of excised pedicels.

The temperature dependence of berry respiration was determined with a water bath held at 10, 20, 30, and 40 °C.

### Pedicel lenticel density

The lenticel density of Chardonnay and Shiraz berry pedicels (stem and receptacle) was assessed using a Nikon SMZ 25 stereo microscope with CCD camera (Nikon Instruments Inc., Melville, NY, USA). Lenticel area (%) was estimated using ImageJ (Schneider *et al.*, 2012) by first adjusting the colour threshold of the image to separate the pedicel from the background and then the lenticels from the pedicel. Subsequently the region of interest (ROI) managing tool was used to estimate the relative area of the pedicels and the lenticels.

### Long-term effect of blocking pedicel lenticels

The pedicel of approximately half of the berries on each cluster of growth chamber-grown Chardonnay were covered with silicone grease at the onset of ripening (first signs of berry softening). Two or three pairs of berries, each pair containing one covered and one uncovered (control) pedicel from one plant, were randomly sampled throughout the course of the experiment at 3, 5, 7, 10, 12, 14, and 18 d after application. Profiles of berry [O_2_] were measured as above, and berries were subsequently assessed for cell vitality (see below). Three pairs of berries were sampled 12 d and 20 d after silicone application and assessed for internal ethanol concentration (see below).

### Berry ethanol concentration

Individual berries were ground to a fine powder under liquid nitrogen. Ethanol was quantified using an Ethanol Assay kit following the manufacturer’s instructions (Megazyme International Ireland Ltd, Wicklow, Ireland). Briefly, alcohol dehydrogenase (ADH) catalysed the oxidation of ethanol to acetaldehyde. Acetaldehyde was then further oxidized to acetic acid and NADH in the presence of aldehyde dehydrogenase (AL-DH) and NAD^+^. NADH formation was measured in a FLUOstar Omega plate reader (BMG LABTECH GmbH, Ortenbery, Germany) at 340 nm.

### Pericarp cell vitality estimation

Cell vitality was estimated using a fluorescein diacetate (FDA) staining procedure on the cut medial longitudinal surface of berries as detailed in Tilbrook and Tyerman (2008) and Fuentes *et al.* (2010). Images were analysed with a MATLAB (Mathworks Inc., Natick, MA, USA) code for determining berry cell vitality (Fuentes *et al.*, 2010). Using ImageJ, the FDA fluorescence signal across the radius at the equator was analysed. The correlation between [O_2_] and fluorescence signal at corresponding distances within Chardonnay and Ruby Seedless berries were examined. The fluorescence signal of growth chamber-grown Chardonnay berries with or without the pedicel covered was also analysed in this way.

### Air spaces within the berry

Chardonnay berries were sampled during the 2015–2016 season for micro-CT, where three berries, each from a different replicate, were used for each sampling time. Grapes were imaged with a Skyscan 1076 (Bruker micro-CT, Kontich, Belgium) at the micro-CT facility at Adelaide Microscopy, where whole berries (pedicel attached) had 2-D projections acquired with 59 kV, 149 µA, Al 0.5 mm filter, 2356 ms exposure, 0.4° rotation step, and 8.5 µm pixel size (equivalent to 15 µm spatial resolution or 3 × 10^–6^ mm^3^ voxel size). NRecon (bruker-microct.com) was used for greyscale image reconstruction. Using CT-Analyer (bruker-microct.com), Otsu thresholding was applied to the volume and despeckle was applied to accept only continuous volume over 500 voxels as connected air spaces. Three-dimensional images of the internal air spaces were generated using CTVox (bruker-microct.com); colour rendering modules were used to distinguish the internal air volume from the berry volume. The 3-D models were then longitudinally sectioned to reveal the internal air space distribution. Quantitative analysis of internal porosity between the berry proximal region and the top (hilum) of the seed(s) was performed by manually selecting the volume of interest and accepting 500 voxels as air spaces.

### Statistical analysis

All data are presented as the mean ±SE. Two-way ANOVA was used for: effect of O_2_ sensor depth and applying N_2_ gas at the point of sensor entry on [O_2_], effect of O_2_ sensor depth and ripening stage on [O_2_], effect of temperature and covering lenticels on respiration, effect of temperature and grape maturity on respiratory Q_10_, effect of covering lenticels and the duration of coverage on [O_2_], TSS, sugar per berry, ethanol, and living tissue profiles. Deming regression was used to determine the association between fluorescent intensity of FDA stain and [O_2_]; this type of regression takes account of error in both *x* and *y* (Strike, 1991). *t*-test was used for differences in: respiration of berry and seed of Chardonnay at two ripening stages, lenticel area on pedicels between Chardonnay and Shiraz, activation energy of O_2_ uptake of Chardonnay and Shiraz berries, and porosity and connectivity index in Chardonnay at two ripening stages. Rates of CD in lenticel-covered berries and control berries were determined using linear regression.

## Results

### Internal oxygen profiles of grape berries

In Chardonnay, [O_2_] decreased from the skin towards the interior of the mesocarp to reach a low concentration at depths of 2.2–4 mm (Fig. 1). The minimum [O_2_] over this depth range was 5.5 ± 5.5 µmol l^–1^. However, with further penetration towards the central axis of the berry, [O_2_] increased and reached a maximum at 7 mm depth (Fig. 1). To test if the [O_2_] profiles were affected by introduced O_2_ via the penetration site, N_2_ gas was gently applied on to the entry point of the sensor during the measurements. The [O_2_] profiles were similar for control and nitrogen-treated berries (Fig. 1), indicating that leakage through the site of penetration did not affect the recorded profiles.

### Changes in internal oxygen profiles and progression of cell death during ripening

**Fig. 2.**
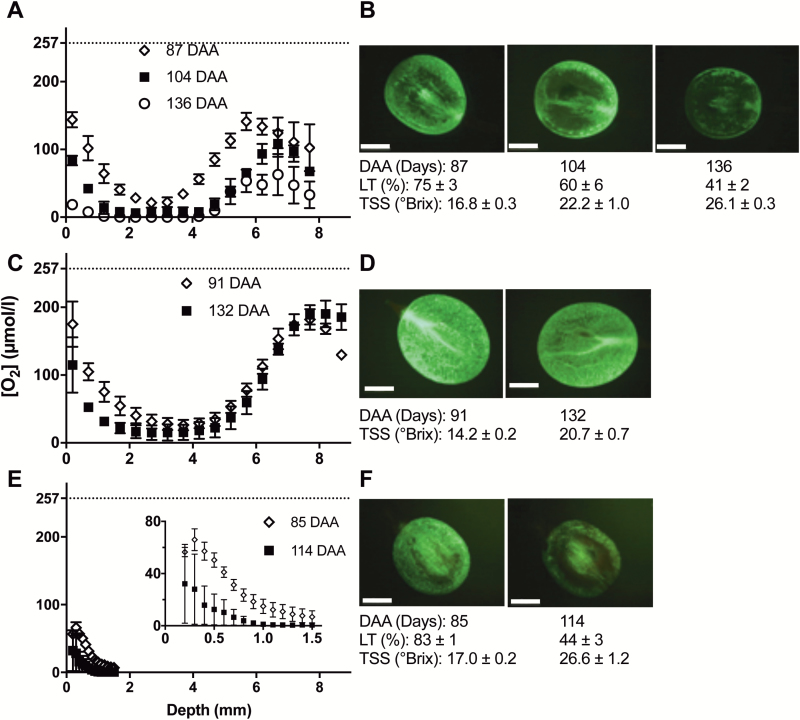
[O_2_] profiles of Chardonnay, Ruby Seedless, and Shiraz berries (A, C, E) at various ripening stages and corresponding examples of living tissue (LT) in the pericarp for each variety (B, D, F). (A) Chardonnay berries were sampled at 87, 104, and 136 DAA in the 2015–2016 season. Two-way ANOVA (repeated) showed that depth accounted for 46.7% of total variation (*P*<0.0001), time accounted for 29.9% of total variation (*P*<0.0001), and interaction accounted for 8.0% of total variation (*P*=0.058). The horizontal dashed line indicates the approximate O_2_ saturation value for Millipore water at room temperature, the same as berries at the time of measurement. (B) Medial longitudinal sections (Chardonnay) stained with FDA, highlighting LT differences at different stages of ripening (corresponding to A). (C) [O_2_] profiles of Ruby Seedless berries sampled at 91 and 132 DAA in the 2016–2017 season. Two-way ANOVA (repeated) showed that depth accounted for 85.2% of total variation (*P*<0.0001), time accounted for 1.2% of total variation (*P*=0.0025), and interaction accounted for 3.7% of total variation (*P*=0.048). (D) LT of Ruby Seedless was close to 100% for the two respective sampling days. (E) [O_2_] profiles of Shiraz berries sampled on 85 and 114 DAA in the 2014–2015 season. Inset shows detail of the profile to 1.5 mm. Two-way ANOVA (repeated) showed that depth accounted for 40.9% of total variation (*P*=0.0005), time accounted for 19.6% of total variation (*P*<0.0001), and interaction accounted for 6.4% of total variation (*P*=0.43). (F) LT of Shiraz. Data are means ±SE, *n*=3 for (A), (C), and (E).

To uncover whether there was a link between the progression of CD and hypoxia within the berry, we determined CD using FDA staining and recorded [O_2_] profiles on berries sampled on the same days. Similar [O_2_] profiles were observed for Chardonnay and Ruby Seedless (Fig. 2A, C), and for Shiraz over the first 1.5 mm (Fig. 2E), but the [O_2_] dropped more steeply across the skins as ripening progressed in all cultivars, resulting in overall lower [O_2_] across the berry. This was manifest as a much lower minimum [O_2_] at the last ripening stage sampled: Chardonnay 0 µmol l^–1^, Ruby Seedless 14.9 ± 8.86 µmol l^–1^, and Shiraz 0 µ µmol l^–1^. Because seeds could not be visualized in Shiraz berries, the micro oxygen sensor could not be moved further into the berry than ~1.6 mm without risking the integrity of the sensor (Fig. 2E). Nevertheless, it was clear that [O_2_] dropped precipitously towards 1 mm (Fig. 2E).

**Fig. 3.**
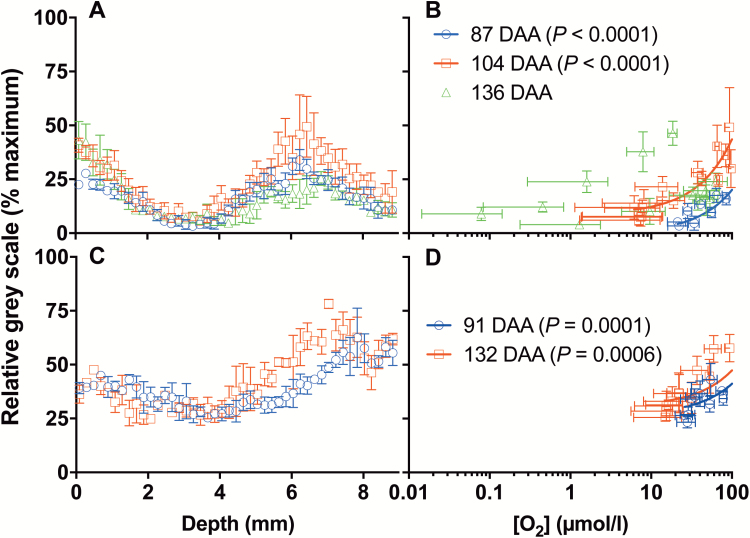
Correlation between living tissue fluorescence signal and [O_2_] profiles. Fluorescence signal [relative grey scale (% maximum)] from FDA stain (high value=higher living tissue) across the radius at the equator of Chardonnay (A) and Ruby Seedless (C). Correlation (Deming regression) between fluorescence signal intensity and [O_2_] at corresponding depths (log scale) in Chardonnay on 87 and 104 DAA (B) and Ruby Seedless on 91 and 132 DAA (D) ([O_2_] profiles shown in Fig. 1). Data are means ±SE, *n*=3.

Vitality staining (Fig. 2B, F) indicated that, for both Chardonnay and Shiraz, CD increased over time as TSS accumulated, and occurred predominantly in the middle of the mesocarp corresponding to the minimum in [O_2_]. Further, the change in fluorescent signal intensity across the radius at the equator of Chardonnay berries showed a similar trend as for berry internal [O_2_] (Fig. 3A), indicating a correlation between cell vitality and internal [O_2_] (Fig. 3B). On the other hand, Ruby Seedless berries maintained cell vitality close to 100% up to 132 DAA, when TSS was 20.7 °Brix (Fig. 2D). While a similar shape of [O_2_] profile was observed within the mesocarp of Ruby Seedless berries when compared with that of Chardonnay berries (Fig. 2C), [O_2_] did not reach zero.

**Fig. 4.**
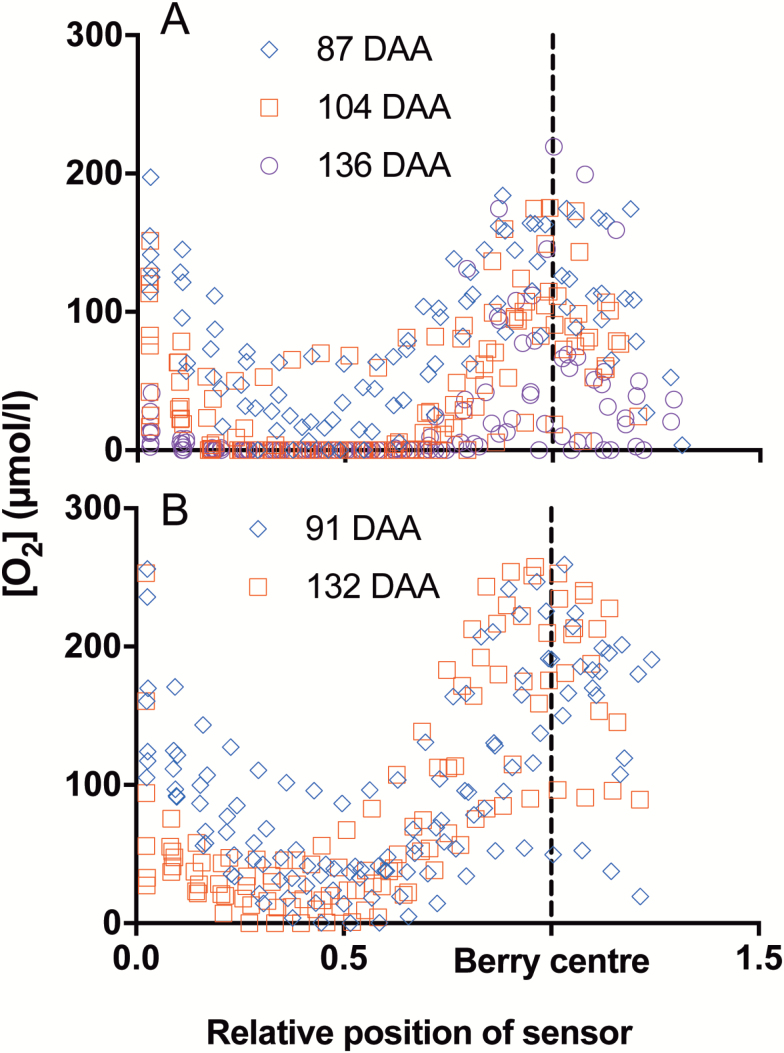
Individual berry [O_2_] profiles normalized to the berry radii. (A) [O_2_] profiles of Chardonnay berries sampled at 87, 104, and 136 DAA in the 2015–2016 season (mean data shown in Fig. 2). (B) [O_2_] profiles of individual Ruby Seedless berries sampled at 91 and 132 DAA in the 2016–2017 season.

Despite the decrease in [O_2_] across the mesocarp during ripening, for Chardonnay and Ruby Seedless berries, [O_2_] started to increase with depth from ~4.2 mm and reached a maximum at ~6.2 mm in Chardonnay and 8.2 mm in the larger Ruby Seedless berries (Fig. 2A, C). Standardizing the position of the sensor relative to the diameter of each berry replicate (Fig. 4) showed that [O_2_] peaked at the central vascular bundle region at all sampling times for both Chardonnay (Fig. 4A) and Ruby Seedless (Fig. 4B).

### Consumption and supply pathways of oxygen within grape berries

**Fig. 5.**
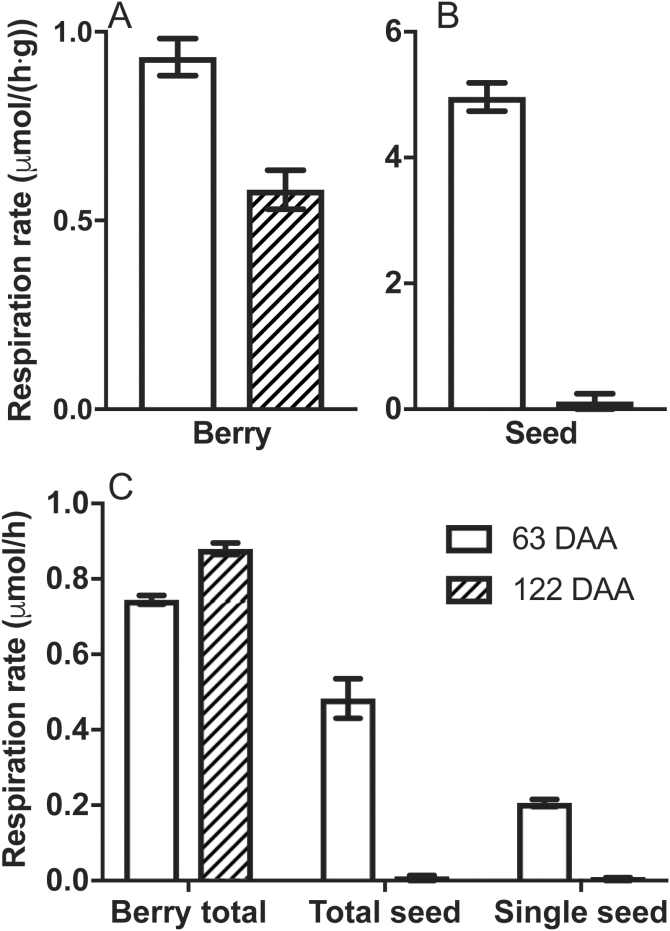
Chardonnay berry and seed respiration (25 °C) at 63 and 122 DAA in the 2015–2016 season. Respiration on a per gram fresh weight basis for berries (A) and seeds (B). (C) Comparison of respiration rates on a per berry basis (including seeds), total seed basis, and single seed basis. Data are means ±SE, *n*=3. All rates are different between 63 and 122 DAA (*t*-test, *P*<0.05).

Considering the link between CD and [O_2_] (Fig. 3), and the lack of CD in well-developed berries of Ruby Seedless (Fig. 2D), we investigated the contribution of seeds to the respiratory demand of the berry in Chardonnay. Seed fresh weight peaks at the beginning of sugar accumulation and skin coloration, with this stage termed veraison (Ristic and Iland, 2005), and was reached ~63 DAA for Chardonnay here. Seed respiration at this stage was 5-fold higher than whole berry respiration on a per gram fresh weight basis. Berry respiration was reduced by about a third at 122 DAA compared with 63 DAA (Fig. 5A); however, seed respiration decreased by 40-fold (Fig. 5B). Berry mass nearly doubled from 7.2 ± 0.5 g at 63 DAA to 13.9 ± 1.4 g at 122 DAA; thus, on a per berry basis, respiration rate increased by ~18% from 63 DAA to 122 DAA (Fig. 5C). The contribution from the total number of seeds in the berry accounted for more than half of the respiratory demand in berries at veraison. This dropped to an insignificant proportion at 122 DAA (Fig. 5C).

**Fig. 6.**
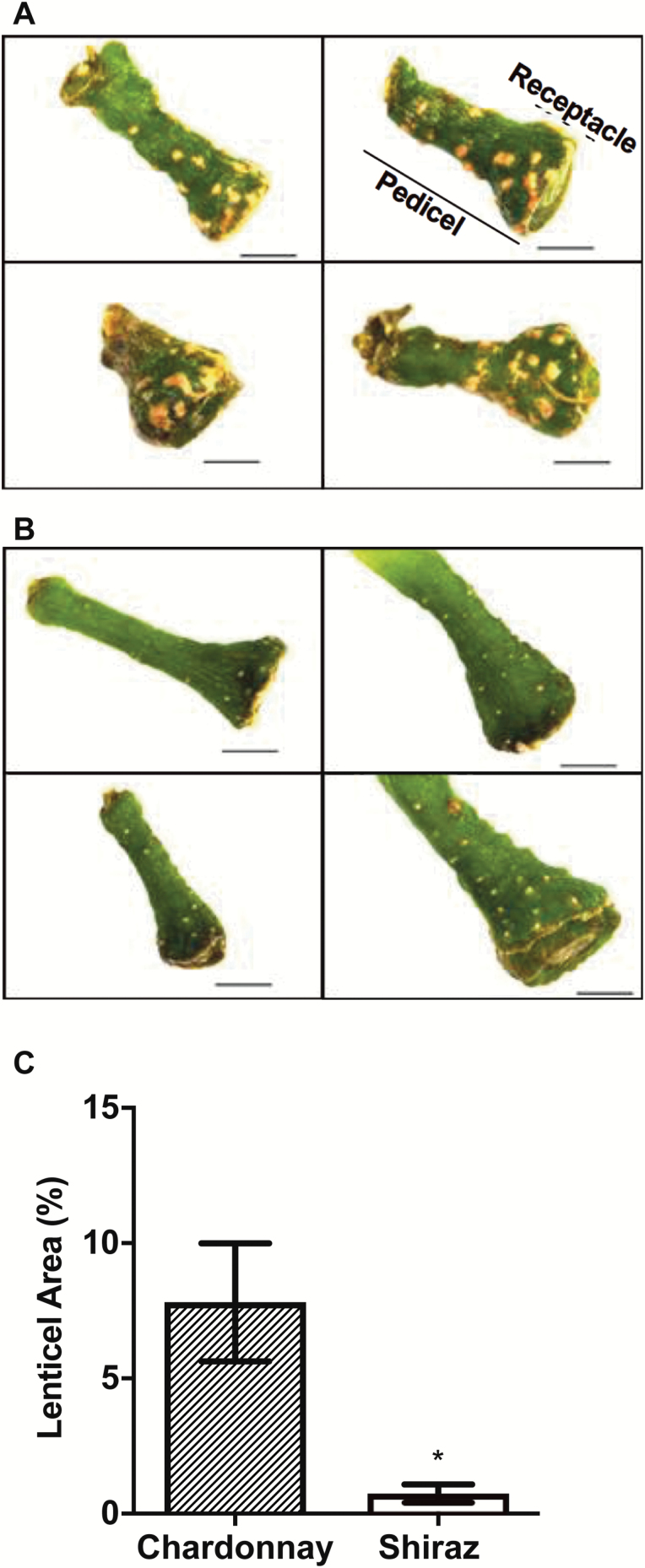
Differences in lenticel morphology and relative lenticel area between Chardonnay (A) and Shiraz (B) berry pedicels. (C) Lenticel area relative to pedicel surface area of Chardonnay and Shiraz berries (chamber grown, 2015) estimated using ImageJ. Scale bars in (A) and (B)=1 mm. Data in (C) are means ±SE, *n*=5, *Significantly different (*t*-test, *P*<0.05).

Differences in resistance to diffusion into the berry may influence the [O_2_] profiles. The pedicel lenticels may offer a pathway for O_2_ entry that could account for the higher concentration towards the central axis of the berry. There were obvious differences in lenticel morphology between Chardonnay (Fig. 6A) and Shiraz berries (Fig. 6B). Individual lenticels on Chardonnay pedicels were larger, and also had a 10-fold larger total surface area as a proportion of pedicel surface area compared with that of Shiraz berries (Fig. 6C).

**Fig. 7.**
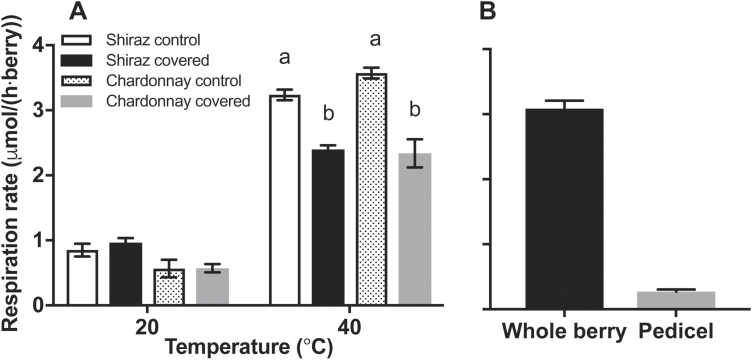
Role of the pedicel in oxygen diffusion as a function of temperature. (A) Respiration of Chardonnay (86 DAA) and Shiraz (77 DAA) berries (per berry basis) at 20 °C and 40 °C with pedicels attached (the 2016–2017 season). Silicone grease covered the lenticels on the pedicel (covered berries). At 20 °C, no significant difference in apparent berry respiration was found between control and pedicel-covered berries for both cultivars. Different lower case letters indicate significant differences between treatments at 40 °C within each cultivar (two-way ANOVA, *P*<0.0001). Shiraz and Chardonnay showed a decrease of 839.7 ± 101.8 nmol h^–1^ and 1377.3 ± 161.3 nmol h^–1^ per berry in respiration at 40 °C (26% and 39% decrease), respectively. (B) Respiration rate of whole berry including attached pedicel and respiration of separated pedicels for Chardonnay at 40 °C. The pedicel accounted for 9% of the whole berry respiration rate. Data are means ±SE, *n*=3.

To determine whether lenticels on the pedicel could be sites for berry gas exchange, respiration was measured on the same batches of berries with or without pedicels covered with silicone grease to impede gas exchange. This was examined at 20 °C and 40 °C as respiratory demand for O_2_ increases with temperature (Hertog *et al.*, 1998). Figure 7A shows that covering the berry pedicel (and lenticels) with silicone grease decreased berry respiration at 40 °C for both Shiraz and Chardonnay berries, but had no effect on respiration at 20 °C. The temperature dependence of respiration was examined in more detail for Chardonnay and Shiraz, with both yielding similar activation energies and Q_10_ (Supplementary Figs S1, S2 at *JXB* online) that did not differ between berries sampled on the two days for each cultivar. The decreased apparent respiration of berries with the covered pedicel was not due to the elimination of pedicel respiration, because the pedicel respiration rate at 40 °C was a small fraction of the total berry respiration (Fig. 7B) and did not account for the decrease observed when pedicels were covered (Fig. 7A), where the decrease in respiration of pedicel-covered Shiraz and Chardonnay at 40 °C was 839.7 ± 101.8 nmol h^–1^ and 1233.9 ± 229.4 nmol h^–1^ per berry.

**Fig. 8.**
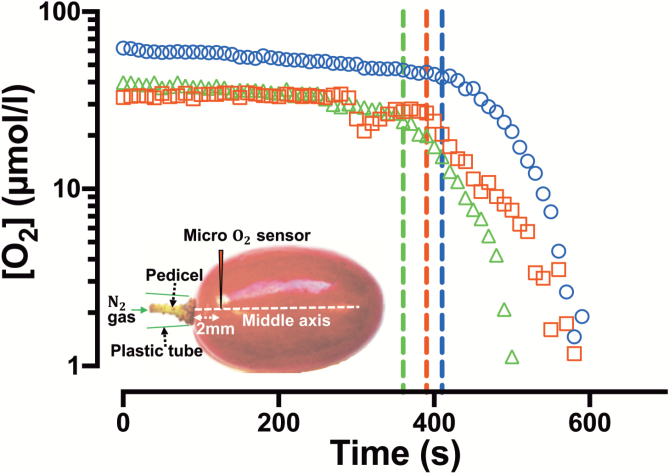
The role of the pedicel in gas diffusion into Ruby Seedless grapes (132 DAA in the 2016–2017 season). [O_2_] of three individual berries as a function of time with the sensor inserted approximately at the central axis of Ruby Seedless ~2 mm from the pedicel. Dashed lines indicate the start of external N_2_ gas delivery over the pedicel. Different symbols indicate different berries. Inset: experimental set-up for applying N_2_ gas over the berry pedicel while measuring [O_2_].

A rapid decrease in [O_2_] was observed at ~2 mm away from the pedicel and close to the centre axis in the Ruby Seedless berries, when an N_2_ stream was activated over the pedicel (Fig. 8).

**Fig. 9.**
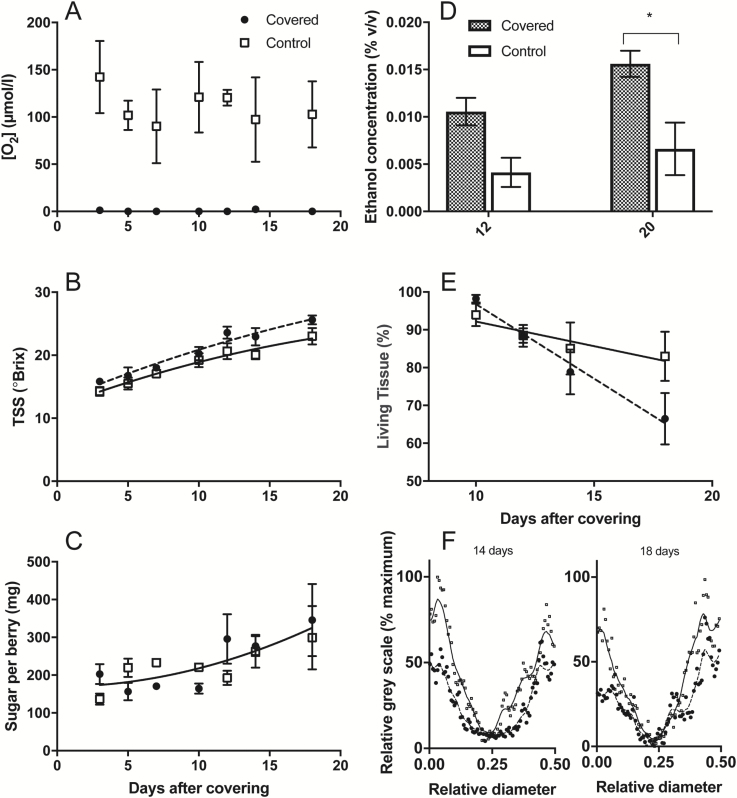
The effect of covering berry pedicels with silicone grease on intact Chardonnay clusters during ripening (chamber grown 2017, open squares=control, filled circles =covered). (A) [O_2_] at the approximate centre axis of berries as a function of time after covering pedicels. Two-way ANOVA showed that covering pedicels reduced [O_2_] (*P*<0.0001). (B) Total soluble solids (TSS) concentration of berries as a function of time after covering pedicels. Pedicel-covered berries showed significantly higher TSS during the course of the experiment compared with control berries (two-way ANOVA *P*=0.003; fits are second-order polynomials, solid line=control, dashed line=covered). (C) Sugar per berry as a function of time after covering pedicels. No significant difference was found between treatments in sugar/berry (combined fit is a second-order polynomial, solid line). (D) Ethanol concentration of berries after 12 d and 20 d with (filled) and without (open) silicone grease covering the pedicels. Two-way ANOVA (Tukey’s multiple comparisons test) showed a significant difference at 20 d after covering (*P*=0.036). (E) Percentage living tissue as a function of time. The slope of the fitted line for covered berries (dashed line) is non-zero (*P*=0.008) and different from the slope of the fitted line (solid line) for uncovered berries (*P*=0.006). (F) Fluorescence signal (FDA stain, relative to maximum, high value=higher living tissue) across the radius at the equator normalized for variation in berry diameter at 14 d and 18 d after covering. Locally weighted scatterplot smoothing fits (LOWESS) are shown for each. Covered (dashed line) versus control (solid line) are significantly different at both times (two-way ANOVA, *P*<0.001). Data are means ±SE, *n*=3, except (F) where SEs are not shown.

An experiment was subsequently conducted using growth chamber-grown Chardonnay vines to test whether covering the pedicel lenticels of berries attached to the vine would affect internal [O_2_] profiles. Three days after covering the berry pedicel with silicone grease, a reduction in [O_2_] at the central vascular region occurred and remained near 0 µmol l^–1^ over the subsequent 15 d (Fig. 9A). For control berries, a maximum of [O_2_] was evident at the central axis across all days of measurement. The concentration of TSS increased with time during the course of this experiment, and was higher for lenticel-covered berries (Fig. 9B). Sugar/berry was not affected by covering the lenticels (Fig. 9C). Ethanol concentration of berries was measured at 12 d and 20 d after covering the pedicel lenticels. These berries, showed higher ethanol content compared with control berries (Fig. 9D), consistent with more fermentation within the hypoxic berries. CD was significantly increased by limiting oxygen diffusion after 10 d of covering the lenticels (Fig. 9E), and this was also evident from examination of transects across the berry (Fig. 9F).

### Air spaces within the grape berry shown by micro-CT

**Fig. 10.**
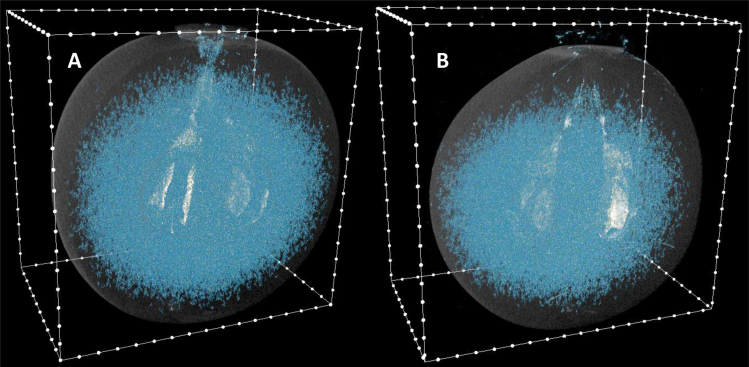
Air spaces in Chardonnay berries as determined by X-ray micro-CT. (A) At 98 DAA (19.3 °Brix) and (B) at 154 DAA (24.5 °Brix) in the 2015–2016 season. Images have been manipulated to indicate the berry outline. Minimum voxel cut-off was 500. White dots on the box outline are at 1 mm intervals.

Using micro-CT, the internal air spaces of Chardonnay berries at two time points during ripening, where air spaces within the berry were greater in total volume than 500 voxels (1.5 × 10^–3^ mm^3^), are shown in Fig. 10. Colour rendering highlighted air space within the berries for both post-veraison (98 DAA, Fig. 10A) and post-harvest (154 DAA, Fig. 10B) berries. Air spaces were connected to the pedicel in the post-veraison berry, but not obviously in the post-harvest berry. It was evident that there were larger air spaces within the locule. Porosity, pores, and channels, between the berry proximal region and seed(s) hilum, did not differ between berries sampled on the two days (Supplementary Fig. S3).

## Discussion

The mesocarp of seeded wine grape berries typically shows a type of programmed CD associated with dehydration and flavour development late in ripening (Tilbrook and Tyerman, 2008; Fuentes *et al.*, 2010; Bonada *et al.*, 2013*b*). Here we show a close similarity between the pattern of CD across the berry mesocarp and [O_2_] profiles where the central regions of the mesocarp had both the highest CD and the lowest [O_2_]. In both Shiraz and Chardonnay, the oxygen deficit in the centre of the mesocarp increased as ripening and CD progressed, essentially becoming anoxic after ~100 d from anthesis under our experimental conditions. This contrasted with the seedless, table grape cultivar where the [O_2_] remained above ~15 µmol l^−1^ (1.1 kPa) in the mid region of the mesocarp, still considered to be hypoxic (Saglio *et al.*, 1988), where CD was less apparent. In our experimental system, however, only three cultivars were tested and there is a confounded effect between cultivar types (wine versus table) with different water and sugar dynamics (Sadras *et al.*, 2008) and between seeded and seedless types. Separating these effects would require the comparison of seeded and seedless isogenic lines. Nonetheless, the strong correlation between CD and [O_2_] profiles, the role of lenticels, seed respiration, ethanol fermentation, and CT images all converge to support our working hypothesis that hypoxia in the mesocarp contributes to CD in the grape berry.

The minimum [O_2_] we measured in the pericarp for both Chardonnay and Shiraz berries (close to zero) may be at or below the *K*_m_ for cytochrome *c* oxidase (0.14 μM) (Millar *et al.*, 1994), and very probably resulted in restricted oxidative phosphorylation and a shift to fermentation as evidenced by the detection of ethanol in Chardonnay berries; testing other cultivars for ethanol production would be of interest. All aerobic organisms require O_2_ for efficient ATP production through oxidative phosphorylation. Lower ATP production occurs under hypoxia when cells shift from oxidative phosphorylation to fermentation (Ricard *et al.*, 1994; Drew, 1997; Geigenberger, 2003). The depletion of ATP has profound consequences on cell physiology, including a change in energy consumption and cellular metabolism (Drew, 1997; Bailey-Serres and Chang, 2005). Loss of membrane integrity responsible for browning disorder in pears is also linked to internal hypoxia and low ATP levels (Saquet *et al.*, 2003; Franck *et al.*, 2007).

Survival of grape berry mitochondria after imposed anaerobiosis (based on succinate oxidation rates) is cultivar dependent, with survival ranging from 1 d to 10 d (Romieu *et al.*, 1992). This work was based on the process of carbonic maceration, a wine-making procedure where whole berries ferment in an anaerobic atmosphere prior to crushing. Ethanol alters the respiratory quotient of grape mitochondria and uncouples oxidative phosphorylation (Romieu *et al.*, 1992). These effects occurred at >1% (volume) ethanol and well above the concentrations we measured in Chardonnay berries (0.015%); however, it is possible that there are locally high concentrations of ethanol within the berry in our case. In a later study, ADH activity and ADH RNA were found to be already high in field-grown Chardonnay berries before anaerobiosis treatment, suggesting that a hypoxic situation already existed in the grapes as a result of some stressful conditions in the field (Tesnière *et al.*, 1993). Our results show that this may be the norm for certain regions within the berry mesocarp and is likely to be exacerbated by high temperature (see below).

The internal [O_2_] of fruit depends on the respiratory demand, and the O_2_ diffusion properties of the skin and internal tissues. These can show genotypic differences as is the case for apple fruit (Ho *et al.*, 2010). In pear fruit, differences in porosity of the cortex, the connectivity of intercellular spaces, and cell distribution may account for variation between cultivars (Ho *et al.*, 2009). For pear it was possible to reconcile the observed variation in gas diffusion with the irregular microstructure of the tissue using a microscale model of gas diffusion. This also appears to be the case for different cultivars of apple as assessed by micro-CT (Mendoza *et al.*, 2007). For grape berries, the [O_2_] profiles in our study would suggest a very low O_2_ diffusivity for the skin since a steep gradient occurred across the skin. Apple skin also showed a very low O_2_ diffusivity and likewise a steep concentration gradient across the skin (Ho *et al.*, 2010). Since sub-skin [O_2_] of grape berries declined dramatically during ripening for all three grape cultivars, it would suggest a decline in O_2_ diffusivity during ripening that may result from the same epidermal and cuticle structural changes that cause a decline in berry transpiration (Rogiers *et al.*, 2004).

Changing properties of the skin, berry porosity and lenticels in the pedicel may all contribute to the reduced internal [O_2_] in grape berries during ripening. Fruit parenchyma can be regarded as a porous medium with air spaces distributed in between the elliptically tessellated cells (Gray *et al.*, 1999; Mebatsion *et al.*, 2006; Herremans *et al.*, 2015). A maximum [O_2_] at the central axis region of both seeded and seedless berries throughout berry development indicates a channel connecting the source of O_2_ intake and the central vascular bundles. Using different approaches, including blockage of pedicel lenticels with silicone grease or applying of N_2_ over pedicels, our experiments demonstrated that the pedicel lenticels are a major pathway for O_2_ diffusion into the grape berry. This corresponds to the predominant air canals observed in micro-CT from the receptacle into the central axis of the berry. Micro-CT to study air space distributions in fruit can reveal important properties that affect gas diffusion (Mendoza *et al.*, 2010; Herremans *et al.*, 2015) as well as internal disorders (Lammertyn *et al.*, 2003). In our work, the visualization of air space connecting the pedicel with the locular cavity around seeds provides the structural link to the measured peaks in [O_2_] around the central vascular region in the berries. This also confirmed the potential O_2_ uptake pathway through the pedicel lenticels, and distribution through the vascular networks. The relatively higher [O_2_] around both central and peripheral vascular bundles may be important for maintaining phloem unloading in the berry, and it is interesting to note that even with severe CD in berries, the vascular bundles generally remain vital (Fuentes *et al.*, 2010). Despite this, we observed higher sugar concentrations in hypoxic berries that had their lenticels covered while still on the vine. This anomaly may be accounted for by decreased water influx because of hypoxia, thereby causing an increase in sugar concentration. Hypoxia is associated with reduced plasma membrane water permeability (Zhang and Tyerman, 1991) caused by closing of water channels of the plasma membrane intrinsic protein (PIP) family (Tournaire-Roux *et al.*, 2003). This is due to sensitivity to lowered cytosolic pH under hypoxia. A PIP aquaporin gene (*VvPIP2;1*) that is highly expressed in the ripening berry (Choat *et al.*, 2009) would be predicted to have reduced water permeation under hypoxia (Tournaire-Roux *et al.*, 2003), perhaps accounting for the decrease in whole berry hydraulic conductance that is consistently observed for Chardonnay and Shiraz (Tilbrook and Tyerman, 2009; Scharwies and Tyerman, 2017).

Lenticels are multicellular structures produced from phellogen that replace stomata after secondary growth (Lendzian, 2006). The impact of lenticels on gas and water permeance compared with periderm of stems has been measured for some species. For *Betula pendula*, the presence of lenticels substantially increased the water permeability of the periderm by between 26- and 53-fold (Schönherr and Ziegler, 1980). Lenticels on the berry pedicel are a preferential site for water uptake for submerged detached berries (Becker *et al.*, 2012). Water vapour and O_2_ permeance of tree phellem with and without lenticels showed that lenticels increased O_2_ permeance much more than that for water, >1000-fold for one species, yet the permeance for water vapour was higher than that for O_2_ (Groh *et al.*, 2002). Interestingly, Schönherr and Ziegler (1980) showed that as the water vapour activity declined (increased vapour pressure deficit), water permeability was strongly reduced. If declining water vapour activity also reduced O_2_ permeability in grape berry lenticels, this could restrict O_2_ diffusion under the very conditions where respiratory demand is increased, namely under water stress and with high temperature and vapour pressure deficit.

The decrease in [O_2_] at the approximate central axis in the seeded Chardonnay berry during development suggests there could be either an increase in respiratory demand, a decrease in the intake of O_2_ via the pedicel lenticels, or decreased porosity through the central proximal axis. Ruby Seedless berries, on the other hand, did not show this reduction. This indicates that there could be structural differences in lenticels between the seeded wine grape cultivar and the seedless table grape, or that the seeds themselves become a significant O_2_ sink (unlikely based on the arguments presented below). The lower lenticel surface area in Shiraz could be indicative of a greater restriction to O_2_ diffusion compared with Chardonnay. Shiraz is well known for its earlier and more rapid increase in CD under warm conditions (Fuentes *et al.*, 2010; Bonada *et al.*, 2013*a*). Unfortunately, it was not possible for us to probe for [O_2_] in the central region of the Shiraz berry to compare with Chardonnay due to not being able to visualize seed position relative to the sensor in Shiraz berries. The role of the pedicel lenticels in allowing grape berries to ‘breathe’ and their variation between cultivars seems to have been overlooked and appears to be unique among fruit. Cluster compactness and pedicel length could also affect the gas diffusion via this passage, ultimately resulting in differences in berry internal oxygen availability throughout ripening.

Another possible explanation for the difference in oxygen profiles between the seeded and seedless cultivars is that seeds are a significant O_2_ sink late in ripening. Oxygen supply to seeds is essential for seed growth, and deposition of protein and oil (Borisjuk and Rolletschek, 2009). On the other hand, low [O_2_] within seeds favours low levels of ROS, thus preventing cellular damage (Simontacchi *et al.*, 1995). The seeded wine grape cultivars Riesling and Bastardo increased O_2_ uptake from <0.45 µmol h^–1^ per berry to ~3 µmol h^–1^ per berry during early ripening, contrasting with seedless Sultana where the maximum O_2_ uptake was 1.5 µmol h^–1^ per berry (Harris *et al.*, 1971). We observed that total seed respiration was more than half of whole berry respiration at around the beginning of ripening. This high O_2_ demand from seeds, prior to the lignification of the outer layer (Cadot *et al.*, 2006), may create a significant O_2_ demand within the berry that could lower [O_2_] in the locule, and potentially lowering the [O_2_] in the mesocarp. However, seed respiration in Chardonnay dramatically declined later in ripening, accounting for the decrease in berry respiration on a per gram basis. During late ripening, [O_2_] in the mesocarp of the seeded cultivar dropped to almost zero. Therefore, it is unlikely that the lower [O_2_] in the mesocarp was caused by a respiratory demand from seeds directly.

Increased temperature advances the onset and increases the rate of CD in Chardonnay and Shiraz berries (Bonada *et al.*, 2013*a*). Using a modelling approach for pear fruit, it was shown that increasing temperature should strongly increase the respiration rate but should not affect the gas diffusion properties, resulting in predicted very low core [O_2_] (Ho *et al.*, 2009). Our direct measures of berry mesocarp [O_2_] profiles concur with this prediction. We also observed typical Q_10_ and activation energy for respiration of 2.47 and 2.27 for whole berry respiration rates between 10 °C and 40 °C for Chardonnay and Shiraz berries, respectively, and it was only at 40 °C that blocking the pedicel lenticels reduced respiration. The activation energies were similar to those reported by Hertog *et al.* (1998) for apple (52 875 J mol^–1^), chicory (67 139 J mol^–1^), and tomato (67 338 J mol^–1^). Unlike pear fruit, wine grape berries ripen on the plant and can become considerably hotter than the surrounding air (Smart and Sinclair, 1976; Tarara *et al.*, 2008; Caravia *et al.*, 2016). Transient high temperatures would create a large respiratory demand and low [O_2_] in the centre of the mesocarp as we observed. However, subsequent cooling during the night or during milder weather will reduce the respiratory demand and increase internal [O_2_] if the diffusivity for O_2_ remains the same. This could then result in production of damaging ROS that may cause unrecoverable cell damage (Pfister-Sieber and Braendle, 1994; Rawyler *et al.*, 2002).

### Conclusion

Grape internal [O_2_] declines during fruit development and is correlated with the profile of mesocarp cell death. Lenticels on the pedicel provide a pathway for O_2_ diffusion into the berry and, when covered to restrict O_2_ diffusion into the berry, cause a large reduction in [O_2_] in the centre of the berry, an increase in ethanol concentration, and cell death. Differences in internal O_2_ availability of berries between cultivars could be associated with seed development and differences in lenticel surface area. The data presented here provide the basis for further research into the role of berry gas exchange in berry quality and cultivar selection for adapting viticulture to a warming climate.

## Supplementary data

Supplementary data are available at *JXB* online.

Fig. S1. Temperature dependence of berry respiration rate.

Fig. S2. Respiratory Q_10_ of Chardonnay and Shiraz berries in response to short-term measurement temperature at two maturity stages.

Fig. S3. Micro-CT analysis of air spaces in Chardonnay berries at two development stages.

Supplementary Figures
