# Supplementary material for: Hypoxia in grape berries: the role of seed respiration and lenticels on the berry pedicel and the possible link to cell death
Source: J Exp Bot. 2018 Mar 6;69(8):2071–83. doi: 10.1093/jxb/ery039 (PMC6018838; doi:10.1093/jxb/ery039)
Supplement: Supplementary Figures [file ery039_suppl_supplementary_figures.pdf]

Hypoxia in the grape berry linked to mesocarp cell death: the role of seed respiration and lenticels on the berry pedicel

Zeyu Xiao, Suzy Rogiers, Victor Sadras, and Stephen Tyerman

SUPPLEMENTARY DATA

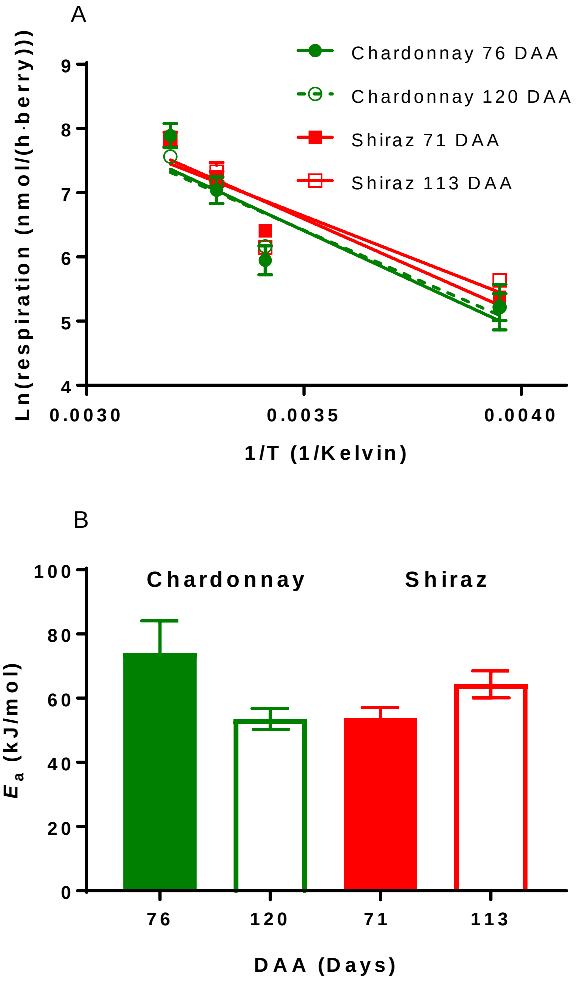

Supplementary Figure 1. Temperature dependence of berry respiration rate. Arrhenius plot of Chardonnay berries sampled on 76 and 120 DAA (11.2 ± 0.9 and 25.5 ± 0.1 °Brix), Shiraz berries sampled on 71 and 113 DAA (11.1 ± 0.4 and 26.2 ± 0.1 °Brix) in 2016-2017 season (A). Slopes (P = 0.98) and intercepts (P = 0.86) were similar between berries sampled, at the two times, within each cultivar. Activation energy of O_2 uptake by the berries (B). Error bars SEM (n = 3).

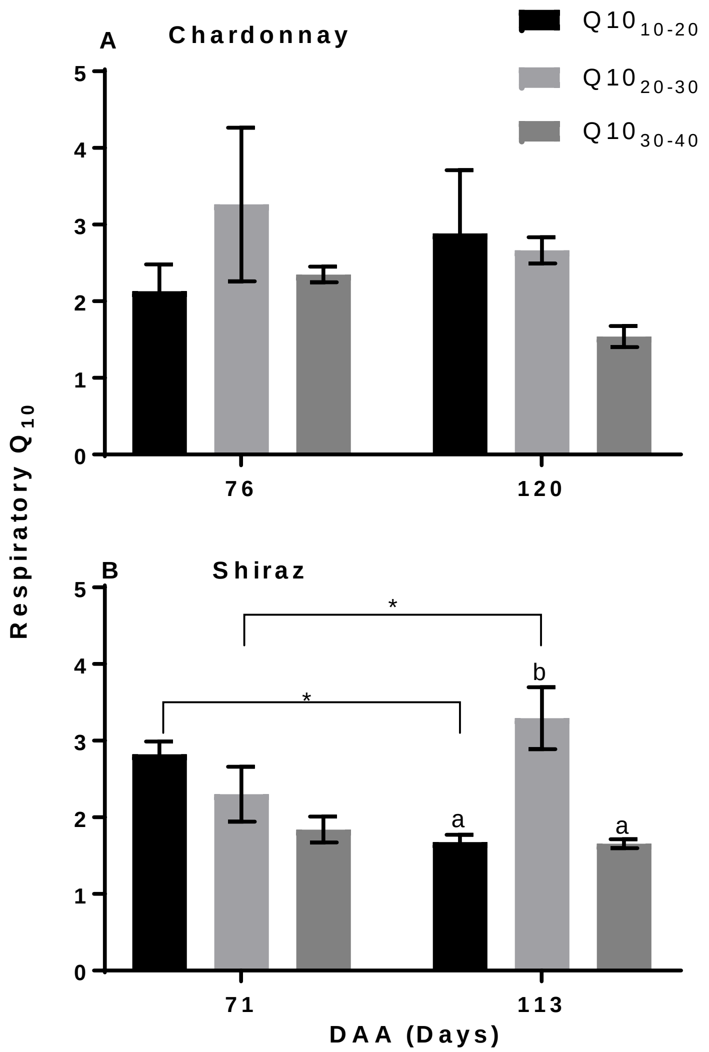

Supplementary Figure 2. Respiratory Q_{10} of Chardonnay (A) and Shiraz (B) in response to short-term measurement temperature and each at two maturity stages (2016-2017 season, Waite vineyards). No difference in the Q_{10} at different temperature classes for Chardonnay berries sampled within the two times were apparent. Q_{10} of Chardonnay berries, sampled at 76 and 120 DAA, at the same temperature classes did not differ from each other. For Shiraz berries sampled at 113 DAA, Q_{10} at 20-30 °C class was higher than the other two temperature classes, difference lower case letters indicate difference (Tukey's multiple comparisons test, $P < 0.05$). At both temperature classes of 10-20 and 20-30 °C, Q_{10} were different between Shiraz berries sampled between 71 and 113 DAA, difference indicated by * (Sidak's multiple comparisons test, $P < 0.05$). Error bars SEM (n = 3).

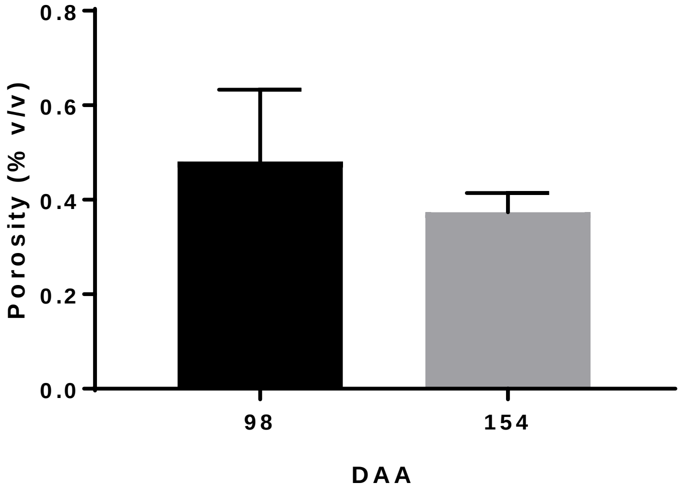

Supplementary Figure 3. Micro CT analysis of porosity, from berry proximal region to the top of seed(s) (hilum), of Chardonnay berries at two development stages (98 DAA, TSS = 18.7 ± 0.7 °Brix; 154 DAA, TSS = 26.8 ± 1.2 °Brix). Error bars SEM (n = 3).
